# Development and Blood–Brain Barrier Penetration of Nanovesicles Loaded with Cannabidiol

**DOI:** 10.3390/ph18020160

**Published:** 2025-01-25

**Authors:** Lucia Grifoni, Elisa Landucci, Giuseppe Pieraccini, Costanza Mazzantini, Maria Camilla Bergonzi, Domenico E. Pellegrini-Giampietro, Anna Rita Bilia

**Affiliations:** 1 Dipartimento di Chimica Ugo Schiff, University of Florence, Via Ugo Schiff 6, Sesto Fiorentino, 50019 Firenze, Italy; lucia.grifoni@unifi.it (L.G.); mc.bergonzi@unifi.it (M.C.B.); 2 Dipartimento di Scienze della Salute, University of Florence, Viale Pieraccini 6, 50139 Firenze, Italy; elisa.landucci@unifi.it (E.L.); costanza.mazzantini@unifi.it (C.M.); domenico.pellegrini@unifi.it (D.E.P.-G.); 3 Centro di Servizi di Spettrometria di Massa (CISM), University of Florence, Viale Pieraccini 6, 50139 Firenze, Italy; giuseppe.pieraccini@unifi.it

**Keywords:** cannabidiol, nanovesicles, Tween 20, hCMEC/D3 cell line, enhanced encapsulation and permeation, stability, controlled release

## Abstract

**Background:** Cannabidiol (CBD) is a highly lipophilic compound with potential therapeutic applications in neurological disorders. However, its poor aqueous solubility and bioavailability, coupled with instability in physiological conditions, significantly limit its clinical use. **Objectives**: This study aimed to develop and characterize nanovesicles incorporating Tween 20 to enhance CBD encapsulation, stability, and the performance across the blood–brain barrier (BBB). **Methods:** Nanovesicles were prepared via thin-film hydration followed by sonication and optimized for size, polydispersity index, and zeta potential. Stability studies were conducted under physiological conditions and during storage at 4 °C. In vitro release studies employed the dialysis bag method, while permeability across the BBB was assessed using PAMPA-BBB and the hCMEC/D3-BBB cell line, characterized for brain endothelial phenotype and largely employed as a model of human blood–brain barrier (BBB) function. Cytotoxicity was evaluated via MTT and LDH assays. **Results:** The quantification of CBD was carried out by HPLC-DAD and HPLC-MS/MS. Nanovesicles with Tween 20 (VS-CBD) exhibited smaller size (65.27 ± 1.27 nm vs. 90.7 ± 0.2), lower polydispersity (0.230 ± 0.005 vs. 0.295 ± 0.003), and higher stability compared to conventional liposomes (L-CBD). VS-CBD achieved high encapsulation efficiency (96.80 ± 0.96%) and recovery (99.89 ± 0.52%). Release studies showed sustained CBD release with Higuchi model fitting (R^2^ = 0.9901). Both PAMPA-BBB and hCMEC/D3-BBB cell lines demonstrated an improved controlled permeability of the formulation compared to free CBD. Cytotoxicity tests confirmed the good biocompatibility of VS-CBD formulations. The addition of Tween 20 to nanovesicles enhanced CBD encapsulation, stability, and controlled release. **Conclusions:** These nanovesicles represent a promising strategy to improve CBD delivery to the brain, offering sustained therapeutic effects and reduced dosing frequency, potentially benefiting the treatment of neurological disorders.

## 1. Introduction

In the past few years, cannabidiol (CBD), a natural non-psychoactive constituent of *Cannabis sativa* L., has emerged as a very attractive compound for its potential anti-inflammatory, anti-oxidative, and neuroprotective effects [1], with possible medical use in several therapeutic areas, including the treatment of ischemia and epilepsy [2,3,4,5].

Despite the plentiful promising clinical uses [6], CBD in oil solution, Epidiolex, is the only medicine on the market based solely on CBD from a natural source [7]. It is designed by EMA as an “orphan medicine” for the treatment of the rare diseases Lennox–Gastaut syndrome, Dravet syndrome, and tuberous sclerosis complex in patients from two years of age [8].

Indeed, CBD has very limited water solubility and extraordinary lipophilicity, which limits its bioavailability (about 6%) [9], when administered in conventional formulations, limiting its clinical efficacy. Cannabidiol is classified as Class II, according to the Biopharmaceutics Classification System (BCS) for oral drugs, as it has very low water solubility and high lipophilicity (12.6 mg/L; log*P* 6.3) [10]. Additionally, its high instability in the stomach acidic medium, a pre-systemic metabolism, and its possible precipitation in the gastrointestinal tract also contribute to its scarce bioavailability [11]. These challenges hinder its clinical application, particularly for diseases requiring precise and sustained drug delivery, such as those targeting the central nervous system (CNS).

A limited number of investigations have reported the tissue distribution of CBD after its administration [12,13]. Preclinical studies have evidenced the presence of CBD or its metabolites (6-OH-CBD and 7-OH-CBD) in the brain after oral dose of 10 mg/kg CBD in different oily formulations. Other routes of administrations, such as subcutaneous and pulmonary ones, result in the decreased distribution of CBD in the brain. Indeed, a few studies have evidenced that diverse CBD formulations might lead to different CBD brain region distribution. Both lipid-free formulations and those based on oils (sesame, coconut, and rapeseed) orally administered at 12 mg/kg led to the highest CBD concentrations in the olfactory bulb, occipital lobe, and striatum. The best performance was obtained with the administration of CBD in lipid-free vehicle (the AUC was 3270 ± 1890 ng/g × h) [14].

The current literature is very limited in terms of selecting the best type of CBD formulation and route of administration to optimize CBD delivery to the brain and its distribution in specific brain regions, but nanovectors have been proposed to optimize the CBD therapy of brain diseases [15,16]. In the present study, nanovesicles were selected for the first time as delivery systems that are able to cross the blood–brain barrier (BBB) and optimize CBD delivery to the brain. In addition, their performance in terms of CBD encapsulation, CBD stability, and permeation were evaluated. Indeed, nanovesicles are very versatile nanoplatforms, which can significantly modify the pharmacokinetics of drugs according to a wide range of lipid composition. Consequently, nanovesicles have an appropriate packing of lipids in the bilayer, suitable average size (from 20 to 200 nm), and proper fluidity of the lipid bilayer, which is an imperative parameter in the context of drug delivery [17,18].

## 2. Results and Discussion

### 2.1. Development and Optimization of CBD Nanovesicles (DLS, ELS, HPLC, and TEM Analysis)

Nanovesicles containing Tween 20 and conventional nanoliposomes were developed by the hydration of the lipid film. The nanovesicle composition was phosphatidylcholine (33 mg/mL), and cholesterol (5 mg/mL) for liposomes (L) and the same composition plus Tween 20 (10 mg/mL) for the nanovesicles containing the edge activator (VS). Two sonication times (2 or 4 min) were applied to reduce nanovesicle sizes and promote system homogenization and stability for a 2-week study at 4 ± 1 °C far from the light. Finally, the formulations were optimized in terms of size, polydispersity, and stability using a sonication of 4 min (Table 1). The size of VS was lower than that of L and both nanovesicles remained stable during the 2 weeks of storage. The PdI of both nanovesicles was stable during storage. The Z-potential of VS was stable during storage, while the Z-potential of L changed during time.

After the optimization of the nanovectors, their encapsulation efficiency (EE%) and recovery (R%) after adding 1 mg/mL of CBD were evaluated for both formulations and are reported in Table 2, together with the physical parameters. The addition of a small amount of CBD in the formulations (1 mg/mL), suitable for the investigation with PAMPA and HCMEC/D3 cell lines, did not alter the physical parameters of the nanovectors with respect to the empty vectors and revealed high EE% and R% for both formulations.

**Figure 1:**
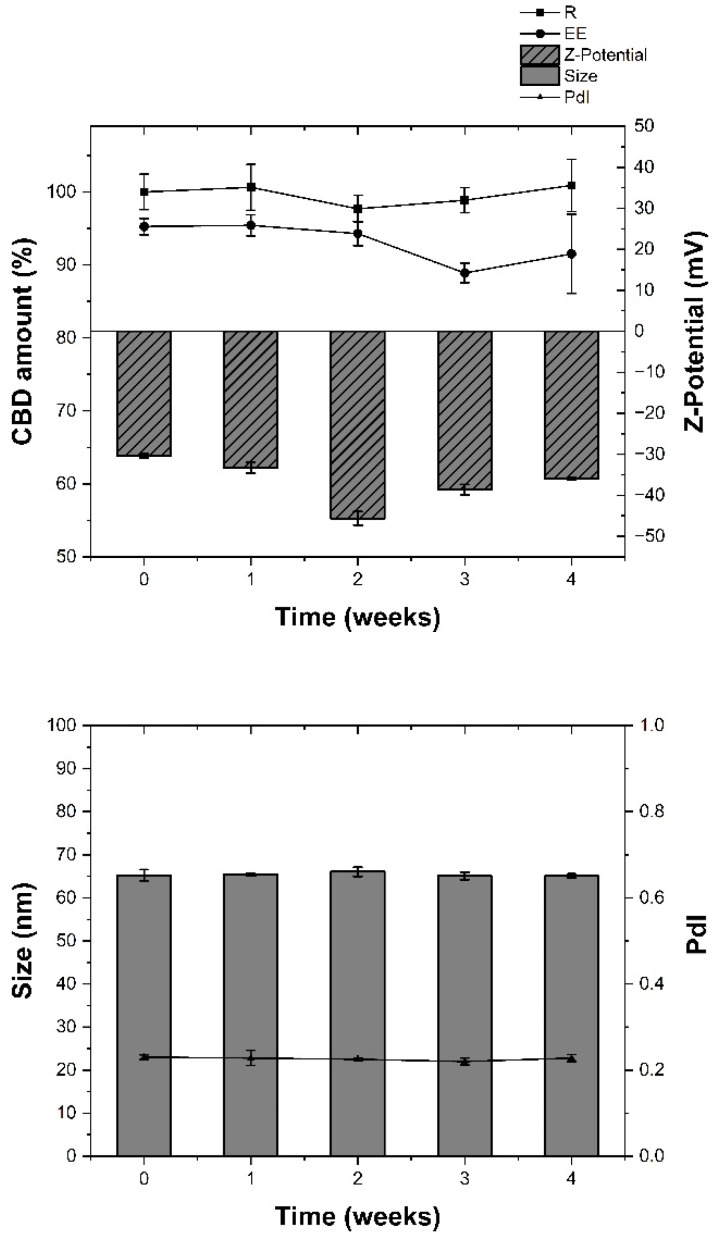
(**Top**) Chemical stability, in terms of recovery percentage (R%) and encapsulation efficiency (EE%), and physical stability, in terms of Z-Potential of VS-CBD stored at 4 ± 1 °C for 1 month. (**Bottom**) Physical stability of VS-CBD stored at 4 ± 1 °C for 1 month. Results are shown as Mean ± SD (*n* = 3).

The physical and chemical properties of VS-CBD were found to remain stable when stored at 4 ± 1 °C far from the light for one month (Figure 1).

Further studies of drug loading were carried out in view of possible forthcoming in vivo investigations of these nanoformulations, by adding to both formulations increasing amounts of CBD up to 10 mg/mL. Only VS-CBD resulted in high EE% (more than 80%) and R% (more than 90%; data not reported). These data agree with the literature, indicating that both Tween 20 and Tween 80 are able to reduce the vesicles’ size and help make small, stable, re-dispersible, and homogeneous nanovectors in addition to optimizing the EE% and release properties [19,20,21].

Surface modification with Tween 20 also influences the fusion of liposomes with cell membranes or their capacity to release contents in response to specific stimuli, which are pivotal for effective drug delivery [22]. Finally, due to the structure of Tween 20 (polyoxyethylene (20) sorbitan monolaurate), a modification of the surface properties of VS with respect to L is expected, thereby creating a hydrophilic barrier that can serve to reduce detectability and adsorption by plasma proteins and immune system components. The presence of polysorbate molecules produces sterically stabilized vesicles, reducing the early clearance of liposomes from the bloodstream and increasing their circulation time, thereby enhancing the capacity to reach and release the drug at specific disease sites [23].

Indeed, a preliminary study supports this hypothesis, demonstrating that the size of VS-CBD remained stable in the presence of bovine serum albumin (BSA) in PBS. The control nanovesicles exhibited a predominant size of 80.0 ± 2.0 nm, with a single, intense peak. Following the addition of BSA, the most intense peak shifted slightly to a size of 86.3 ± 4.1 nm. Alongside this shift, a new peak was observed at approximately 10 nm, likely corresponding to free albumin in solution [24].

The small increase in the hydrodynamic diameter of the nanovesicles after BSA addition suggests minimal interaction between the nanovesicles and the protein. The presence of the new peak further supports the coexistence of albumin in the system, potentially interacting weakly with the nanovesicles. These observations are consistent with the effects of ionic strength and pH of the PBS buffer, which are known to influence particle size measurements [25].

**Figure 2:**
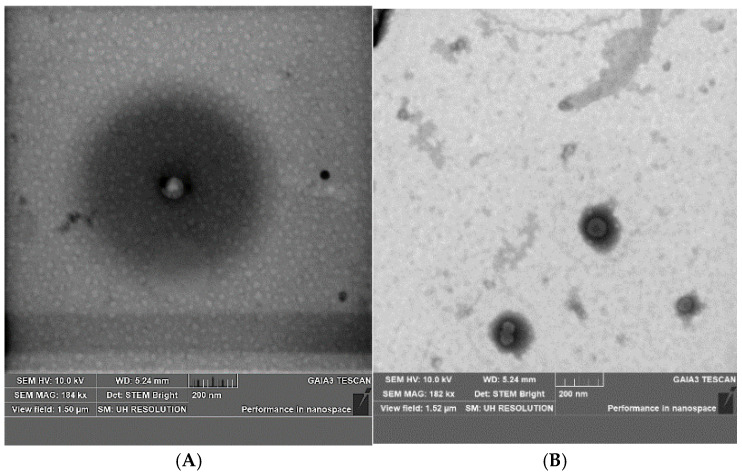
Pictures of (**A**) VS and (**B**) VS-CBD, bar = 200 nm, obtained by STEM analysis.

Finally, both VS and VS-CBD were examined by transmission and scanning electron microscopy (STEM) at 200 nm magnification (Figure 2), to assess if sizes were consistent with data from DLS.

### 2.2. In Vitro Release Studies and Stability Studies

For the in vitro release study, a suitable solution was selected to obtain CBD in sink conditions. A solution of methanol in PBS (pH 7.4) in a ratio of 30:70 was selected. The solubility of CBD in this solution was about 10 µg/mL.

The volume of the acceptor was chosen to be three- to ten-fold greater than the volume required to dissolve the CBD amount in the bag. In the release study, the performance of VS-CBD was compared with the unformulated CBD (FREE-CBD), which was obtained by dissolving CBD in methanol and then diluting with PBS to achieve the same solution of the release medium with a concentration of 1 mg/mL.

**Figure 3:**
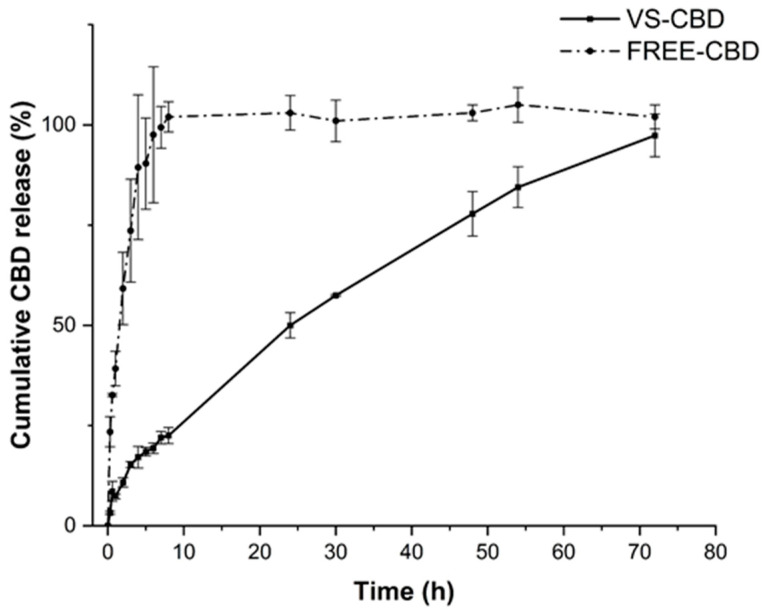
Release profiles of CBD from VS-CBD and FREE-CBD (CBD dispersion in MeOH and PBS).

FREE-CBD was a stable and uniform dispersion with particle sizes of approximately 600 nm as evidenced by DLS analysis. The release profile of FREE-CBD showed an initial burst release, which is consistent with CBD diffusion through the membrane (Figure 3). By contrast, the VS-CBD release profile was controlled and sustained, highlighting the ability of the vesicular structure to modulate the release rate of CBD over an extended period (Figure 3). Indeed, the absence of a burst release reduces the likelihood of CBD reaching harmful levels in the bloodstream and suggests a sustained therapeutic effect by maintaining a consistent release of CBD over time, which enhances the overall efficacy without the need for frequent dosing. Furthermore, this controlled release can improve patient compliance by reducing the frequency of administration, obtaining a more convenient therapeutic option. These behaviours provide significant advantages in maintaining a steady, effective, and safe release profile, which is essential for long-term therapeutic applications of CBD.

In addition, the release data of VS-CBD were fitted with various kinetic models, after 8 h and 70 h of release. The Higuchi model showed an excellent fit, both after 8 and 70 h of release (Table 3). The Higuchi model is a typical kinetic release model through the diffusion process based on Fick’s law, i.e., square root time-dependent, evidencing that the nanovesicles act as a barrier that regulates the CBD’s diffusion, preventing the rapid initial release observed in the dispersion. In general, drug release from liposomes depends on kinetic factors like drug permeability and thermodynamic parameters such as drug partitioning across the bilayer surface. These modelling approaches are very important for the formulator to optimize the vesicles for improve the therapeutic approaches because the kinetic release studies can optimize the in vitro–in vivo correlation [26].

There are many studies evidencing that the release of drugs from liposomes obeys the Higuchi diffusion model. Indeed, a liposomal chitosan gel base (1%, m/m) loaded with 5-fluorouracil (5-FU) for topical application evidenced that 5-FU was related to the bilayer composition and conditions of hydration [27]. In another study, multilamellar liposomes loaded with 5-FU also complied with Higuchi’s square root model [28].

### 2.3. Diffusion Behaviour Through Artificial Membrane (PAMPA-BBB)

PAMPA offers researchers a quick, inexpensive method of evaluating the permeability of test compounds, pre-formulation excipient screening, and the performance of a new formulation. The assay has been recently validated by testing about 2000 small molecules to correlate in vitro PAMPA-BBB data with in vivo brain permeation data in rodents. A correlation of 77% suggested that models developed using data from PAMPA-BBB can forecast in vivo brain permeability [29].

The PAMPA-BBB experiment, conducted over 18 h at 25 °C, provided valuable insights into the passive diffusion of CBD dispersion (FREE-CBD) across a membrane, simulating its ability to cross the blood–brain barrier (BBB) without the involvement of active transport mechanisms. The free dispersion showed an effective permeability (P_e_) value comparable to that of progesterone, with a slightly higher retention in the system. This suggests that FREE-CBD can effectively diffuse across the membrane, but a considerable fraction is retained, confirming its affinity for the lipid bilayer.

The VS-CBD formulation showed a significantly lower P_e_ value when compared to that of progesterone and FREE-CBD due to the encapsulation of CBD in the bilayer and consequently modulating the diffusion of CBD, thereby preventing its immediate and uncontrolled passage through the membrane (Table 4). Indeed, the nanovesicles effectively regulated the release of CBD, ensuring more gradual diffusion compared to FREE-CBD. This controlled release profile is critical for therapeutic applications where sustained and predictable drug delivery is desired and helps to reduce the risk of toxicity from the rapid burst release of the drug.

### 2.4. Cytotoxicity and Cell Viability on HCMEC/D3 Cell Line

**Figure 4:**
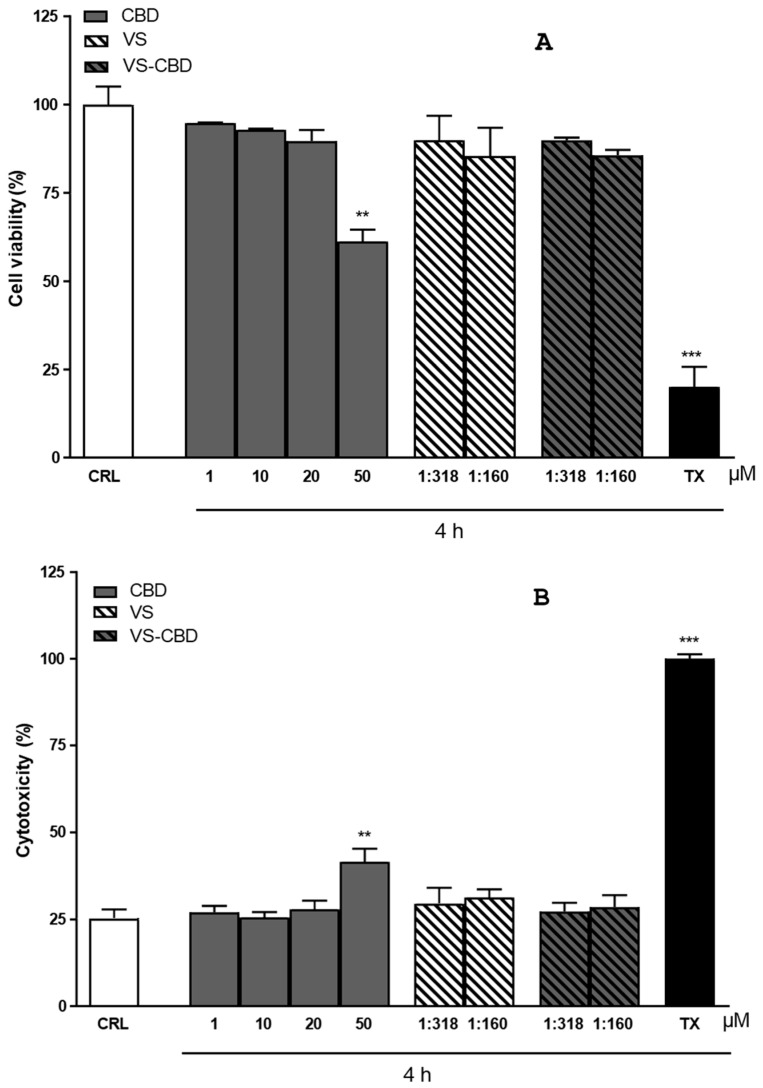
(**A**) Percentage of viable cells and (**B**) cytotoxic assessments FREE-CBD, VS, and VS-CBD. Results are shown as Mean ± SEM (*n* = 4). ** *p* < 0.01; *** *p* < 0.001.

Cytotoxicity and cell viability were initially tested on FREE-CBD. Toxicity was observed from a concentration of 50 µM upwards, which was found to be toxic (Figure 4A). The formulation was also tested, both empty and loaded with CBD, after dilution to achieve CBD concentrations of 10 and 20 µM (dilution 1:318 and dilution 1: 160, respectively). The resulting VS and VS-CBD concentrations were statistically comparable to negative controls, as reported in Figure 4A (percentage of viable cells) and Figure 4B (cytotoxic assessments). Neither the unloaded nanovesicles nor VS-CBD with CBD concentrations of 10 µM (VS-10) and 20 µM (VS-20) were cytotoxic and the cell viability was suitable. For this reason, these two nanovesicles were selected for further permeation studies.

### 2.5. CBD Permeability Evaluation Across HCMEC/D3 Cell Line

The hCMEC/D3 brain microvascular endothelial cell line provides a model for the human BBB, exploring the drug transport mechanisms. The cells retain the expression of most transporters and receptors present in vivo within the human BBB, which is characterized by tight junctions with extremely high electrical resistivity. This cell line is very useful for the evaluation of permeability of drugs and their drug delivery systems across the BBB [30,31].

The apparent permeability coefficient (P_app_) of hCMEC/D3 correlates well with in vivo permeability data, and thus permeability studies were conducted to predict the permeability of FREE-CBD and VS-CBD across the BBB. NaF was employed as the negative control to monitor the integrity of the cell layer by measuring its P_app_.

**Figure 5:**
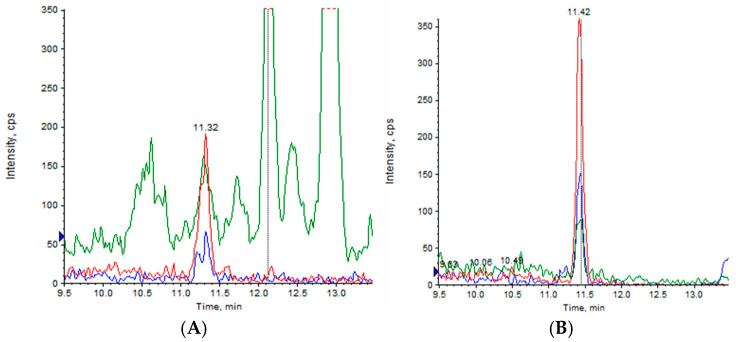
(**A**) Injection of a blank (methanol) and (**B**) injection of empty nanovesicles at least 80 times more concentrated than the samples under analysis. The mass spectrometer acquired positive ion signals in multiple reaction monitoring mode by recording three fragment ions of the protonated CBD ion (*m*/*z* 315.3), at 259.0 (blue line), 193.1 (red line), and 134.9 *m*/*z* (green line).

For the measurement of CBD permeability across the HCMEC/D3 cell line, an HPLC-MS/MS method was developed to ensure accurate and reliable results. The analysis evaluated all potential interferences, ensuring data quality. A scan of the samples revealed that there were no detectable adducts with salts, confirming that the samples were clean and interference-free (Figure 5). The mass spectrometer acquired positive ion signals in multiple reaction monitoring mode by recording three fragment ions of the protonated CBD ion (*m*/*z* 315.3), at *m*/*z* 259.0 (blue line), *m*/*z* 193.1 (red line), and *m*/*z* 134.9 (green line), which represent the typical fragmentation pattern of CBD, as previously reported.

Indeed, besides the molecular ion at *m*/*z* 315.3, the fragment at *m*/*z* 259.0 originates from the loss of four carbon units from the terpene moiety. The fragment at *m*/*z* 193.1 corresponds to olivetol with the carbon unit attached to C2 of the benzene ring. The fragment at *m*/*z* 134.9 matches the terpene moiety [32,33].

In addition, an analysis of the empty nanovesicles was conducted to prevent the overestimation of CBD content in nanovesicle donor and lysate samples. VS produced a higher signal when diluted much less than the run samples, suggesting that its contribution is minimal and comparable to background noise.

Regarding the matrix effect, a solution of 2.90 µg/mL CBD was diluted in a ratio of 3:1 with buffer or methanol, followed by serial dilutions with ultrapure water. The following table (Table 5) summarizes the effects of buffer concentration on the ratio of the areas of methanol (A_MeOH_) and buffer (A_buffer_).

These results suggest that with decreasing buffer concentration, the ratio between methanol and buffer signal changes, indicating the potential influence of the buffer on the detection of CBD at various concentrations. This effect must be considered when analyzing permeability results to ensure accuracy in the quantification of CBD.

Finally, three different calibration curves were developed to account for the varying conditions of the samples analyzed: one calibration curve was prepared for the undiluted samples in buffer (acceptor compartment), ensuring that the quantification of CBD in these samples would be accurate without dilution artifacts affecting the results; a second calibration curve for the cell lysate; and a third calibration curve for the donor solutions from the permeability experiment (Table 6).

By generating these three distinct calibration curves, we ensured the precise and reliable quantification of CBD across the different sample types, compensating for any matrix or dilution effects that could have influenced the analytical results. This method allowed us to accurately measure the CBD concentrations and permeability in each condition with confidence. The characteristics of the curves are reported in Table 6.

**Figure 6:**
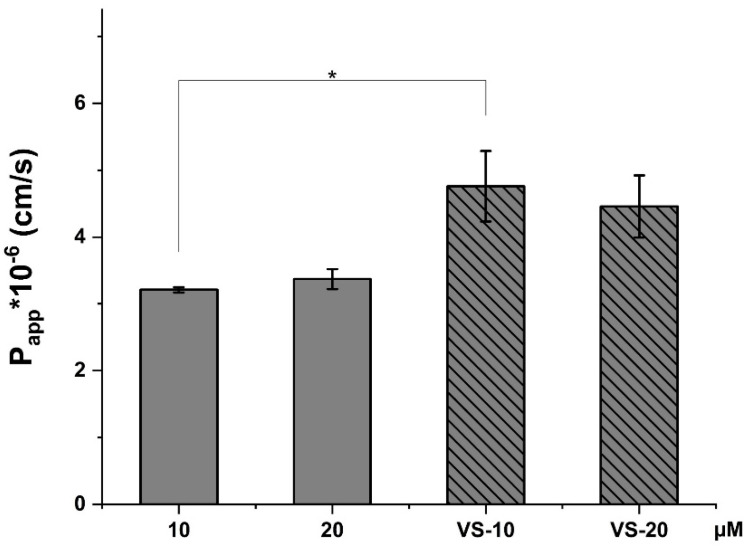
Apparent permeability (P_app_) values for CBD solutions at concentrations of 10 µM and 20 µM, as well as VS-CBD (VS-10 and VS-20). * *p* < 0.05.

The P_app_ value (Figure 6) for FREE-CBD at 10 µM is lower compared to the 20 µM solution, suggesting that higher concentrations of free CBD enhance its permeability through the HCMEC/D3 monolayer.

However, both VS-10 and VS-20 (nanovesicle-encapsulated CBD) show higher P_app_ values compared to the free CBD solutions, indicating that nanovesicle encapsulation enhances the permeability of CBD. This suggests that the nanovesicles help CBD cross the cellular barrier more effectively.

**Figure 7:**
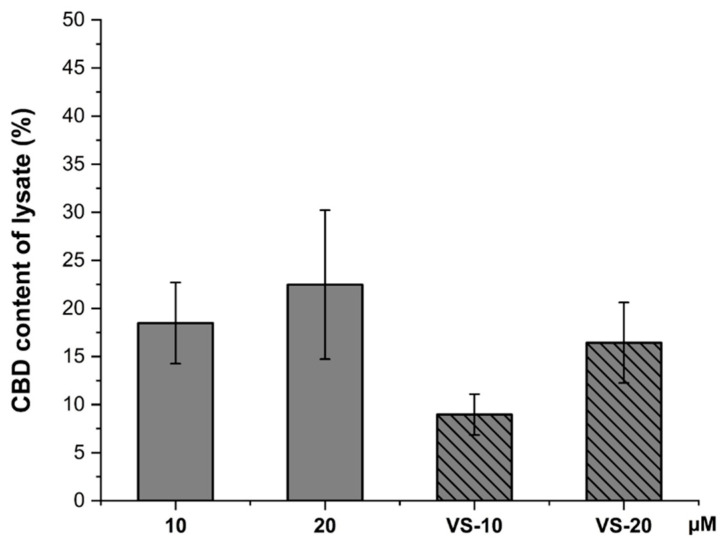
CBD content extracted from lysate (%) for CBD solutions at concentrations of 10 µM and 20 µM, as well as VS-CBD (VS-10 and VS-20).

Figure 7 shows the CBD content in the cell lysate, representing the amount of CBD that has been taken up by the cells.

Interestingly, the CBD solution at 20 µM resulted in the highest cellular uptake, with around 20% CBD content in the lysate, while the 10 µM solution showed a lower uptake.

On the other hand, the VS-10 and VS-20 formulations showed lower CBD content in the lysate compared to the free CBD solutions. This suggests that while nanovesicles enhance permeability, they might reduce direct cellular uptake of free CBD, possibly due to the controlled release or slower release of CBD from the nanovesicles into the cells.

**Figure 8:**
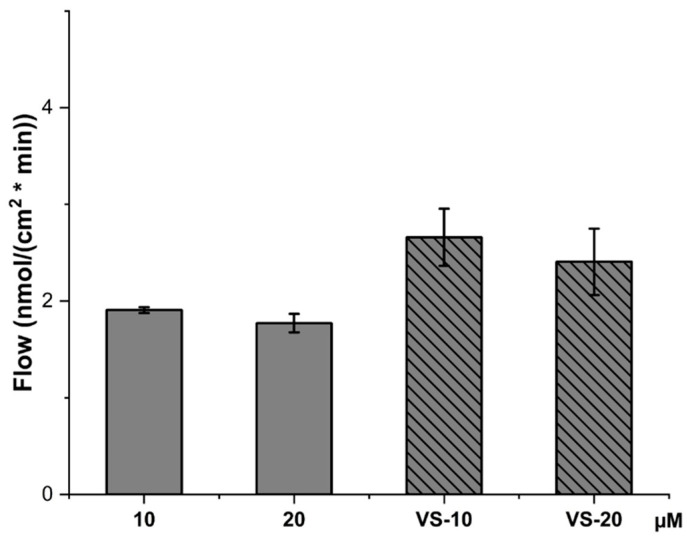
Flow rate across cell monolayer for CBD solutions at concentrations of 10 µM and 20 µM, as well as VS-CBD (VS-10 and VS-20).

Figure 8 presents the flow rate of CBD across the HCMEC/D3 monolayer. Like the P_app_ values, the flow rate is higher for VS-10 and VS-20 compared to the free CBD solutions, further confirming that nanovesicle encapsulation improves the overall flow of CBD across the cellular layer.

The higher flow rates for the nanovesicle formulations highlight the nanovesicles’ role in enhancing the transport of CBD across the endothelial monolayer, possibly due to their ability to interact with the cell membrane more effectively or due to their structural properties that facilitate better transport across the barrier.

The data suggest that the VS-CBD formulations significantly improved the permeability and flow of CBD across the HCMEC/D3 cell line compared to free CBD solutions. While the free CBD solutions (especially at 20 µM) showed higher direct uptake into the cells (as seen in the lysate content), the nanovesicle formulations enhanced the overall transport through the cellular barrier, likely due to their ability to modulate the release and interaction with the membrane, remaining intact at the end of the experiment.

This controlled and enhanced transport of CBD via nanovesicles is particularly important for therapeutic applications where the efficient crossing of biological barriers, such as the blood–brain barrier, is necessary. While cellular uptake is reduced, nanovesicles may provide a more sustained release, maintaining therapeutic levels of CBD over time without an initial burst or excessive accumulation within cells, which could reduce potential toxicity.

## 3. Materials and Methods

### 3.1. Materials

Cannabidiol (CBD, 98% *w*/*w*) from a natural source was purchased from Galeno Srl (Prato, Italy). Phospolipon 90G (P90G) was purchased from Lipoid AG (Cologne, Germany) with the support of its Italian agent AVG srl. Methanol, acetonitrile, and formic acid of HPLC grade, CH_2_CL_2_ and MeOH at pharmaceutical grade, cholesterol, Tween 20, sodium fluorescein, and salt phosphate buffer were from Sigma Aldrich (Milan, Italy). Hanks’ balanced salt solution (HBSS) buffer was from Thermofisher scientific (Monza, Italy). Ultrapure water was produced using a synergy UV Simplicity water purification system provided by Merck KGaA (Molsheim, France).

### 3.2. Liposome and Nanovesicle Preparation

Conventional liposomes and nanovesicles were prepared as previously reported [34]. Briefly, Phospholipon 90G (33 mg/mL) and cholesterol (5 mg/mL) were dissolved in a 3:1 mixture of MeOH and CH_2_CL_2_ and evaporated using a rotary evaporator for 25 min at 35 °C.

The hydration phase was conducted at 37 °C for 30 min using ultrapure water or a 10 mg/mL solution of Tween 20. To produce small unilamellar nanovesicles from multilamellar nanovesicles, an ultrasonic probe was used until 4 min (with pulsed duty cycles of 2 s on and 2 s off, amplitude 40%) with the sample in an ice bath to prevent lipid degradation. Finally, a centrifugation of 2 min at 2000× *g* was performed to remove any metallic particles potentially released by the ultrasound probe within the liposomal dispersion.

### 3.3. Dynamic and Electrophoretic Light Scattering

The physical properties of the nanovesicles were determined by the light scattering technique, allowing the calculation of the mean hydrodynamic diameter (nm), polydispersity index (PdI), and zeta potential (mV) of the nanovesicles. Dynamic and electrophoretic light scattering (DLS-ELS) analyses were performed using the Zetasizer Pro Red from Malvern Panalytical (Alfatest Srl, Milan, Italy), equipped with a He-Ne gas laser with a maximum output power of 10 mW and a beam wavelength of 632.8 nm, and an avalanche photodiode (APD) detector. The measurements were performed at 25 °C with a backscatter detection angle of 173°. All data were processed using the cumulants method as defined in the international standard ISO22412 as previously reported [34]. Measurements were performed in triplicate at 25 ± 2 °C and a scattering angle of 90°. Samples were analyzed after a 10-fold dilution in ultrapure water, using square polystyrene cuvettes for DLS and folded capillary zeta cells for ELS.

### 3.4. Scanning Transmission Electron Microscopy (STEM)

A scanning electron microscope (Gaia 3, Tescan s.r.o., Brno, Czech Republic) was used to evaluate the size and architecture of nanovesicles. It is a focused ion beam scanning electron microscope (FIB-SEM) operated in high vacuum mode with an electron beam voltage of 20 kV and a bright field transmission electron microscope detector. Gaia 3 was equipped with an EDS X-ray microanalysis system (EDAX, AMETEK, Berwyn, PA, USA), TEAM EDS Basic Software Suite TEAM™, and was delivered with a STEM (scanning transmission electron microscopy) detector, which provides a complementary method for the image acquisition of transmitted electrons. The detector consists of multiple semiconductor sensors, which are used for both bright-field and dark-field imaging. The transmitted electron signal can be collected by positioning the detection system underneath the sample. The instrument is located in a room that is kept at a constant temperature of 19 degrees Celsius. Prior to image acquisition, the formulation was placed on a copper grid covered with a carbon film. The excess sample was then blotted off the grid with filter paper, resulting in the formation of a thin film that was stained with a phosphotungstic acid solution (1% *w*/*v*) in distilled water. Analysis was performed three minutes after staining according to previous studies [35].

### 3.5. Quantification of CBD

#### 3.5.1. HPLC-DAD and HPLC-FLD

An HPLC 1200, coupled to a diode array detector (DAD) and a fluorescence detector (FLD), allowed the recording of the full UV-VIS absorption spectrum and fluorescence, respectively, of the molecules eluting from the column. An EclipseXDB-C18 chromatographic column (4.6 × 150 mm, 3.5 μm) was used for the quantification of CBD and sodium fluorescein (NaF). The selected analytical method includes a mobile phase of acidic water (pH = 3.2 with formic acid) and acetonitrile, flowing at a rate of 0.8 mL/min for 35 min, with a timeline described in Table 1. A post run of 10 min was used to re-equilibrate the column (Table S1).

For CBD, the chromatographic profile was recorded at 225 nm for CBD. For NaF, λ_ex_ was set at 460 nm and fluorescence was detected at 515 nm (λ_em_, green).

A calibration curve was constructed by dissolving CBD and NaF in MeOH to a concentration of 1 mg/mL for both substances. The stock solution was diluted 20, 200, and 600 times and these solutions were used to construct the calibration curve with injection volumes of 3 and 5 µL for FD20, 3, 5, and 7 µL for FD200, and 3 µL for the FD600 solution.

This calibration line, with a correlation coefficient R^2^ of 0.999966815 for CBD and 0.99995153 for NaF, was then used for all subsequent quantitative analyses.

#### 3.5.2. HPLC-MS/MS

Analyses were performed by liquid chromatography coupled to tandem mass spectrometry on a system consisting of a Perkin Elmer Series 200 liquid chromatograph (Perkin Elmer, Milan, Italy) complete with autosampler and column oven coupled to a Sciex 4000 Q trap mass spectrometer with electrospray interface (V-spray) operating as a triple quadrupole.

The chromatographic separation was carried out on a Restek Ultra AQ C18 column (Restek srl, Cernusco sul Naviglio Milan, Italy), 100 × 2 mm, 3 µm, maintained at 40 °C, with elution in gradient according to Table S2. The flow rate was 0.3 mL/min.

The mass spectrometer acquired positive ion signals in multiple reaction monitoring (MRM) mode by recording three fragment ions of the protonated CBD ion (*m*/*z* 315.3), at 259.0, 193.1, and 134.9 *m*/*z*.

The dwell time was 200 ms for each transition. Data processing was performed using Analyst software version 1.6.3.

The matrix effect was studied using a 2.90 ug/mL stock solution of CBD in MeOH and diluted 1:3 with methanol or Hank’s balanced salt solution (HBSS), and both solutions were diluted by successive 1:2 dilutions in water.

### 3.6. Recovery and Encapsulation Efficiency

The encapsulation efficiency (EE%) of CBD entrapped into the bilayer’s liposomes was calculated using the direct method, expressed as a percentage of the number of substances initially used in the liposomal preparation. The non-encapsulated CBD was removed by means of dialysis. In total, 1 mL of liposomal suspension was transferred into a dialysis bag (cut-off 6–8 kDa), which was stirred in 1 L of water at room temperature for one h. The EE% was calculated using the equation below (Equation (1)):

(1) $$EE\%=\frac{CBD\;experimentally\;found\;after\;the\;purification\;(mg)\;}{Weighted\;CBD\;(mg)}\;*\;100$$

The recovery percentage (R%) was evaluated by using the same procedure but without the initial purification step and was calculated using the following equation (Equation (2)):

(2) $$R\%=\frac{CBD\;experimentally\;found\;(mg)\;}{Weighted\;CBD\;(mg)}\;*\;100$$

The content of CBD within liposomes was quantified by HPLC-DAD analysis. The disruption of the liposomes was achieved by the addition of methanol (dilution factor = 100), followed by sonication for 15 min. Ultracentrifugation was performed for 5 min at 14,000 rpm.

### 3.7. CBD Release from VS-CBD

In vitro release experiments were conducted at 37 °C for up to 72 h using the bag dialysis method. A volume of 500 µL was encapsulated in Spectra/Por dialysis bags made of regenerated cellulose with a molecular weight cut-off of 6–8 KDa. The receptor compartment was filled with 200 mL of a solution consisting of methanol in PBS (pH 7.4) in a ratio of 30:70. Withdrawals (1.5 mL) were performed at 0.3, 0.6, 1, 2, 4, 6, 8, 24, 30, 48, 54, and 72 h. This setup was designed according to the solubility principle to ensure sink conditions for CBD diffusion.

The percentage of CBD release (%) was evaluated from nanovesicles and from a dispersion of CBD in the same buffer as the recipient compartment. The dispersion was prepared by dissolving CBD in MeOH, followed by the addition of PBS.

All samples were analyzed by the HPLC-DAD method.

The kinetic and mechanism of CBD release from the liposomes were evaluated by selecting the optimal model based on the highest regression coefficient value for the obtained data. The data were fitted using the zero-order, first-order, Higuchi, and Korsmeyer–Peppas models [36].

### 3.8. PAMPA-BBB Assay

The adapted protocol for performing the experiment was taken from Radan and coworkers [37]. Briefly, the lipid membrane for the PAMPA assay was prepared by dissolving 20 mg of porcine polar brain lipid (PBL) in 1 mL of dodecane, and 5 μL of the prepared solution was used to coat the filter membrane. Progesterone and unformulated CBD (FREE-CBD) were dissolved in DMSO (5 mg/mL) and then diluted in physiological phosphate buffer (PBS, pH = 7.40) to obtain a final concentration of 50 μg/mL. Liposomes were diluted 20-fold to obtain the same concentration.

Effective permeability (P_e_) was calculated as described below:

(3) $$P_{e}=\frac{-2.303V_{d}}{A\left(t-\tau_{lag}\right)}\left(\frac{1}{1+r_{v}}\right){log}_{10}1-\left(\frac{1+r_{v}^{-1}}{1-R}\right)\frac{C_{a}(t)}{C_{d}(0)}$$

(4) $$V_{a}C_{a}\left(t\right)+V_{d}C_{d}\left(t\right)=V_{d}C_{d}(0)(1-R)$$

where C_d_(t) and C_a_(t) are the concentrations (µM) of the compound in donor and acceptor wells at time t, respectively; t is the incubation time (s); C_d_(0) is the concentration of the compound in the donor well at time 0 (µM); V_d_ (0.25 cm^3^) and V_a_ (0.30 cm^3^) are the volumes of donor and acceptor wells (cm^3^); A (25 cm^2^) is the filtration area; τ_lag_ is the time needed to saturate the membrane (approximately 20 min); R is the mole fraction of compound retained by the membrane; and parameter r_v_ is calculated with the formula r_v_ = V_d_/V_a_.

### 3.9. Stability Studies

A first preliminary study was conducted to evaluate the stability of VS-CBD after incubation in PBS buffer containing BSA to mimic the in vivo conditions. Briefly, 100 µL of nanovesicle dispersion was exposed to 1 mL of a BSA solution at a concentration of 40 mg/mL in PBS, chosen to represent the physiological plasma albumin levels. The samples were maintained at 37 °C and agitated at 500 rpm for 8 h to simulate blood flow and the dynamic interactions between the formulations and plasma proteins [38].

In addition, the stability of nanovesicles was evaluated over a period of one month. The samples were kept at 4 ± 1 °C and their physical and chemical stability was monitored at fixed time intervals: the physical stability was checked by monitoring the sizes, the polydispersity index, and the ζ-potential, while the chemical stability was determined by quantifying the R% and EE% by HPLC-DAD analysis.

### 3.10. MTT and LDH Assays

The cell viability after free CBD and VS-CBD exposure in hCMEC/D3 cell was assessed by MTT and LDH assays [39]. The formulations and free CBD were incubated at different concentrations for 4 h. A part of the medium of each treatment was taken and preserved for the LDH assay, and cells were incubated with MTT at the concentration of 1 mg/mL. Finally, DMSO was used to dissolve MTT formation and absorbance was recorded at 550 and 690 nm. HBSS was used as a positive control and Triton X-100 as a negative control, and the cell viability was expressed as a percentage compared to the cells incubated only with HBSS. The extent of cell death after free CBD and VS-CBD exposure was quantitatively evaluated by measuring the amount of LDH release from injured cells. The LDH level corresponding to complete cell death was obtained in the presence of triton X-100 (positive control). All the values were expressed as a percentage compared to TX release.

### 3.11. Permeability Through hCMEC/D3 Cell Line

The blood–brain barrier hCMEC/D3 cell line (product number: SCC066; catalogue no.: 9QQ0M9) was procured from EMD Millipore Corporation (Burlington, MA, USA).

The hCMEC/D3 cell line monolayers were used as a permeability assay for FREE-CBD and VS-CBD. Fluorescein sodium salt (NaF) at a concentration of 10 µg/mL was used as an integrity control marker, given its known permeability coefficient (P_app_) for this cell line.

FREE-CBD and VS-CBD were prepared to achieve CBD concentrations of 10 and 20 µM (VS-10 and VS-20, respectively). To obtain these concentrations VS-CBD were diluted 160- and 318-fold using HBSS, respectively. FREE-CBD solutions were prepared by diluting a 5 mg/mL CBD stock solution in DMSO with HBSS.

The donor compartment, acceptor compartment, and cell lysates from each well were analyzed by HPLC-MS. Different treatments were applied to each sample type: acceptor samples were directly injected, lysates were diluted 3-fold, and donor samples were diluted 5-fold. For all lysates, CBD was extracted by placing liposomes in an ultrasonic bath for 30 min, followed by centrifugation at 5000 rpm for 20 min. The liposome donors were ultrasonicated for 15 min, followed by centrifugation at 14,000 rpm for 10 min.

Apparent permeability (P_app_) was evaluated as follows:

(5) $$P_{app}\left(\frac{cm}{s}\right)=\frac{V_{d}}{A\;*\;M_{D}(0)}\;*\;\frac{∆M_{A}(t)}{∆t}$$

where V_d_ is represents the volume of the donor compartment, M_D_ and M_A_ are the concentrations of the donor compartment at time 0 and the receiver compartment after 4 h incubation, respectively, and A is the permeation area (1.13 cm^2^).

The flow rate of CBD across the monolayer was expressed (Equation (6)) as:

(6) $$Flow\left(\frac{mmol}{{cm}^{2}\;*\;min}\right)=\frac{{mmol}_{a}}{A\;*\;t}$$

where mmol_a_ is the amount of CBD in the receptor compartment (mmol) after 4 h incubation.

And the CBD content of lysates was expressed as:

(7) $$CBD\;content\;of\;lysate\;\left(\%\right)=\frac{CBD\;in\;the\;lysate\;(nmol)}{total\;amount\;of\;CBD\;(nmol)}\;*\;100$$

## 4. Conclusions

According to the literature, vesicles provide significant advantages in managing drug diffusion, ensuring sustained release, and improving the overall pharmacokinetics of drugs, especially in complex biological environments such as the BBB. CBD is a very lipophilic drug and can cross the BBB but due to significant limitations of stability after administration in conventional drug delivery systems, there is an urgent need to develop suitable nanovectors for its delivery to the brain. This study is the first report on CBD loaded in nanovesicles. The addition of Tween 20 to conventional liposomes made of phosphatidylcholine and cholesterol enhanced both the loading and stability of CBD. Developed nanovesicular systems can achieve more predictable and effective drug delivery, particularly in therapeutic areas such as brain diseases, which require controlled drug administration. The studies of PAMPA-BBB and HCMEC/D3 permeability and the in vitro release experiments clearly support this behaviour. In particular, the PAMPA-BBB experiment, designed to evaluate the passive diffusion through a membrane mimicking BBB, supports the differentiation between FREE-CBD and VS-CBD. Indeed, FREE-CBD exhibited higher permeability in the PAMPA-BBB model, resulting in higher Papp values and flow rates observed in the HCMEC/D3 permeability study. This study suggests that FREE-CBD can easily cross both synthetic and biological membranes, driven by concentration gradients, but it also reflects the lack of control over the diffusion process. Such behaviour may be suitable for rapid drug delivery but presents limitations in achieving sustained and controlled therapeutic levels, especially for applications where precise delivery is required, as in the case of BBB. The study on HCMEC/D3 cell line revealed significant differences in the behaviour of FREE-CBD versus VS-CBD. These findings provide valuable insights into the mechanisms by which both free and encapsulated CBD diffuses through biological barriers and highlight the advantages of the use of controlled release systems like nanovesicles. The developed nanovesicles can avoid excessive early uptake, while promoting steady diffusion over time, which could ensure more predictable therapeutic outcomes and reduce the risks associated with burst release. While this can be advantageous for achieving a quick therapeutic effect, it poses challenges for maintaining controlled drug levels and may increase the risk of adverse effects due to rapid accumulation in target cells or tissue. Conversely, VS-CBD provides a controlled release profile, characterized by lower initial uptake but efficient permeability over time, which is crucial especially for chronic diseases with long-term care with a reduced number of daily doses, as in many brain diseases.

In addition, nanovesicles have a lipid bilayer structure that mimics biological membranes, allowing them to interact more effectively with cell membranes. This interaction enhances the overall transport across the membrane, even though the release of the encapsulated drug is slower. The nanovesicles can fuse or interact with the membrane, facilitating the internalization of their content, which explains the higher flow rate observed.

Additionally, nanovesicles provide protection to the encapsulated CBD, preserving its integrity and preventing premature degradation or interaction with membrane components. This protective effect helps maintain the drug’s bioavailability, ensuring that more CBD is available for transport across the membrane. Nanovesicles may also affect paracellular transport, temporarily altering the integrity of cell junctions and increasing overall diffusion through cellular layers. Moreover, nanovesicles can improve local solubility near the membrane, which enhances the release and transport of CBD compared to free CBD, which might suffer from solubility challenges in aqueous environments. This work provides a comprehensive report on the use of CBD-loaded nanovesicles for BBB penetration and highlights the potential of these nanovesicular systems to improve therapeutic outcomes for brain diseases. A possible limitation for the industrial scalability of the developed nanovesicles, with the aim of a clinical application, could be related to the need of a sonication process to optimize sizes and PdI. However, many are the possible innovative industrial manufacturing methods of vesicles, in particular microfluidic production, which can overcome critical scale-up limits [40]. Concerning the dialysis process to remove the non-encapsulated CBD, the results evidenced that the recovery was about 99.9% versus 96.8% of encapsulation efficiency, and the irrelevant difference in these two values does not influence the performance of VS-CBD. Accordingly, a dialysis process is not essential.

Future studies, particularly in vivo investigations, are warranted to further validate these findings and explore their translational potential in clinical applications.

## Figures and Tables

**Table 1:** Physical properties of liposome (L) and nanovesicle containing Tween 20 (VS) and stability after 2 weeks of incubation. Results are shown as Mean ± SD (*n* = 3).

	Tween 20 (mg/mL)	Time Sonication (min)	Time: 0			Time: 2 Weeks		
			Size (nm)	PdI	Z-Potential (mV)	Size (nm)	PdI	Z-Potential (mV)
L	0	4	90.7 ± 0.2	0.295 ± 0.003	−43.35 ± 2.25	96.28 ± 0.3	0.291 ± 0.003	−28.35 ± 2.25
VS	10	4	67.7 ± 0.8	0.288 ± 0.008	−32.13 ± 0.800	65.7 ± 1.0	0.263 ± 0.002	−33.15 ± 0.700

**Table 2:** Physical and chemical characteristics of liposomes (L) and nanovesicles (VS) loaded with CBD. Results are shown as Mean ± SD (*n* = 3).

	Tween 20 (mg/mL)	CBD (mg/mL)	Size (nm)	PdI	Z-Potential (mV)	R %	EE %
L-CBD	-	1	70.1 ± 2.3	0.181 ± 0.011	−56.20 ± 7.561	96.89 ± 1.52	91.80 ± 0.76
VS-CBD	10	1	65.27 ± 1.27	0.230± 0.005	−30.31 ± 0.54	99.89 ± 0.52	96.80 ± 0.96

**Table 3:** Fitting parameters and coefficients of determination (R^2^) for different kinetic models applied to the release profile of CBD from VS-CBD. The models evaluated include zero-order, first-order, Higuchi, and Korsmeyer–Peppas. Data are reported after 8 h and 70 h.

Kinetic Model	Function	R^2^
	**After 8 h**	
Zero-order	y = 2.56x + 4.56	0.9052
First-order	y = −0.01x + 1.98	0.9232
Higuchi	y = 8.14x + −0.10	0.9815
Korsmeyer–Peppas	y = −0.07x + 1.96	0.8465
	**After 70 h**	
Zero-order	y = 1.37x + 9.80	0.9642
First-order	y = −0.02x + 2.02	0.9331
Higuchi	y = 11.92x + −5.41	0.9901
Korsmeyer–Peppas	y = −0.44x + 2.05	0.8516

**Table 4:** Effective permeability (P_e_), recovery percentage (R%), and mass retention (MR%) for progesterone (highly permeable standard), NaF (Sodium fluorescein, a non-permeable molecule), VS-CBD, and FREE-CBD; Results are shown as Mean ± SD (*n* = 4). Statistical analysis on P_e_ values was performed at *p* < 0.05 level. Letters indicate different groups according to Tukey’s test.

	P_e_ × 10^−6^ (cm/s)	R%	MR%
Progesterone	2.29 ±0.51 ^A^	87.47 ± 0.06	12.53 ± 0.06
FREE-CBD	1.90 ± 0.03 ^A,B^	72.82 ± 0.01	27.16 ± 0.01
VS-CBD	1.11 ± 0.01 ^B^	100.95 ± 0.02	/
NaF	/	100.31 ± 2.30	/

**Table 5:** Ratio between the areas obtained by the injection of a 2.90 µg/mL solution of CBD, subsequently diluted 3:1 with Hanks’ balanced salt solution (HBSS) buffer or methanol, followed by serial dilutions with ultrapure water.

Ratio (AMeOH/Abuffer)	Buffer in Solution (% *v*/*v*)	CBD (ng/mL)
1.56	80	725
1.65	40	362.5
1.24	20	181.2
0.98	10	90.6

**Table 6:** Curves developed to evaluate CBD permeation across the HCMEC/D3 cell line, reflecting the sample treatment. The concentration range for all curves was between 0 and 3500 ng/mL.

Sample Analyzed	% MeOH	Correlation	Slope	Intercept
Time 0	100	0.9998	0.000131323	1.21
Donor	80	0.9998	0.000130587	−3.12
Lysate	33	0.9997	0.000131504	0.93
Acceptor	0	0.9998	0.005018337	0.55

## Data Availability

Data is contained within the article or Supplementary Materials.

## Supplementary Materials

The following supporting information can be downloaded at: https://www.mdpi.com/article/10.3390/ph18020160/s1, Table S1: Elution method via HPLC-DAD-FLD for the quantification of CBD and NaF; Table S2: Elution method via HPLC-MS for the quantification of CBD.
