# Supplementary material for: Development and Blood–Brain Barrier Penetration of Nanovesicles Loaded with Cannabidiol
Source: Pharmaceuticals (Basel). 2025 Jan 25;18(2):160. doi: 10.3390/ph18020160 (PMC11859449; doi:10.3390/ph18020160)
Supplement: Supplementary file 1 [file pharmaceuticals-18-00160-s001.zip › pharmaceuticals-3411604-supplementary.pdf]

Table S1. Elution method via HPLC-DAD-FLD for the quantification of CBD and NaF.

Time (min)	Acetonitrile (%)	Acid Water (pH 3.2)
0.1	20	80
2	25	75
10	50	50
11	50	50
20	99	1
35	20	80

Table S2. Elution method via HPLC-MS for the quantification of CBD.

Time (min)	Acetonitrile (%)	Acid Water (pH 3.2)
0.1	25	75
0.5	25	75
8	100	0
14	100	0
15	25	75
20	25	75
